# Transcriptional activity of *PIF* and Pong-like Class II transposable elements in Triticeae

**DOI:** 10.1186/s12862-017-1028-6

**Published:** 2017-08-03

**Authors:** Dragomira N. Markova, Roberta J. Mason-Gamer

**Affiliations:** 1 Department of Biological Sciences, University of Illinois at Chicago, M/C 067 840 West Taylor Street, Chicago, IL 60607 USA; 2 Present address: Department of Plant Sciences (mail stop 3), 151 Asmundson Hall, University of California, Davis, CA 95616 USA

**Keywords:** *PIF*-like, Pong-like, Transposable elements, DNA transposon, Class II, Transcription, Phylogeny, Triticeae

## Abstract

**Background:**

Transposable elements are major contributors to genome size and variability, accounting for approximately 70–80% of the maize, barley, and wheat genomes. *PIF* and Pong-like elements belong to two closely-related element families within the *PIF*/Harbinger superfamily of Class II (DNA) transposons. Both elements contain two open reading frames; one encodes a transposase (ORF2) that catalyzes transposition of the functional elements and their related non-autonomous elements, while the function of the second is still debated. In this work, we surveyed for *PIF*- and Pong-related transcriptional activity in 13 diploid Triticeae species, all of which have been previously shown to harbor extensive within-genome diversity of both groups of elements.

**Results:**

The results revealed that *PIF* elements have considerable transcriptional activity in Triticeae, suggesting that they can escape the initial levels of plant cell control and are regulated at the post-transcriptional level. Phylogenetic analysis of 156 *PIF* cDNA transposase fragments along with 240 genomic partial transposase sequences showed that most, if not all, *PIF* clades are transcriptionally competent, and that multiple transposases coexisting within a single genome have the potential to act simultaneously. In contrast, we did not detect any transcriptional activity of Pong elements in any sample.

**Conclusions:**

The lack of Pong element transcription shows that even closely related transposon families can exhibit wide variation in their transposase transcriptional activity within the same genome.

**Electronic supplementary material:**

The online version of this article (doi:10.1186/s12862-017-1028-6) contains supplementary material, which is available to authorized users.

## Background

Triticeae is a pooid tribe with approximately 30 genera and 300–400 species [1], including wheat, barley, and rye. The tribe’s economic importance has made it the focus of many evolutionary and genetic studies over the last few decades. The Triticeae genome is large and complex, with approximately 70–80% composed of transposable elements (TEs) [2–7].

Eukaryotic TEs have been divided into two main groups based on their structure and transposition mechanism. Class I TEs (retrotransposons) transpose by reverse transcription of an RNA intermediate, while Class II TEs (DNA elements) transpose via a double-stranded DNA intermediate through a “cut and paste” mechanism whereby the element is excised and reinserted elsewhere in the host genome. These usually have terminal inverted repeats (TIRs) whose size and sequence are characteristic of the family or superfamily to which the element belongs. Autonomous Class II elements encode all functional products required for transposition, including a transposase gene (TPase) that catalyzes DNA cleavage and transposition. Non-autonomous elements are usually deletion derivatives of autonomous elements that only retain the terminal sequences necessary for recognition and activation by the transposition machinery of autonomous elements [8, 9]. All TE superfamilies contain both autonomous and non-autonomous elements [10].

Transposable elements are major contributors to genome size and variability, and gene evolution [11–16]. Their ability to move and amplify within a genome results in mutational activity that can alter gene structure and function [17, 18] through loss of genes [12, 14, 15], changes in expression levels [19], or evolution of new functions [20–22]. Once integrated in the genome, some TEs accumulate mutations and become transcriptionally and/or transpositionally inactive [23–26]. A fine balance between transcription, transposition, and host survival should be reached, and the host tightly controls the activity of TEs [27]. However, despite mutation and cell control, some TEs remain transcriptionally and transpositionally active [28–34].

This work is focused on the transcriptional activity of a superfamily of Class II elements called *PIF*/Harbinger in the genomes of 13 diploid species from the wheat tribe, Triticeae. The *PIF*/Harbinger elements form a widespread superfamily of DNA transposons, which consists of *PIF* and Pong-like elements. *PIF* and Pong-like elements were first discovered in the maize [34] and rice [30] genomes, respectively, and they have since been detected in the genomes of many flowering plants, animals, and fungi [28, 35–38].

**Fig. 1:**
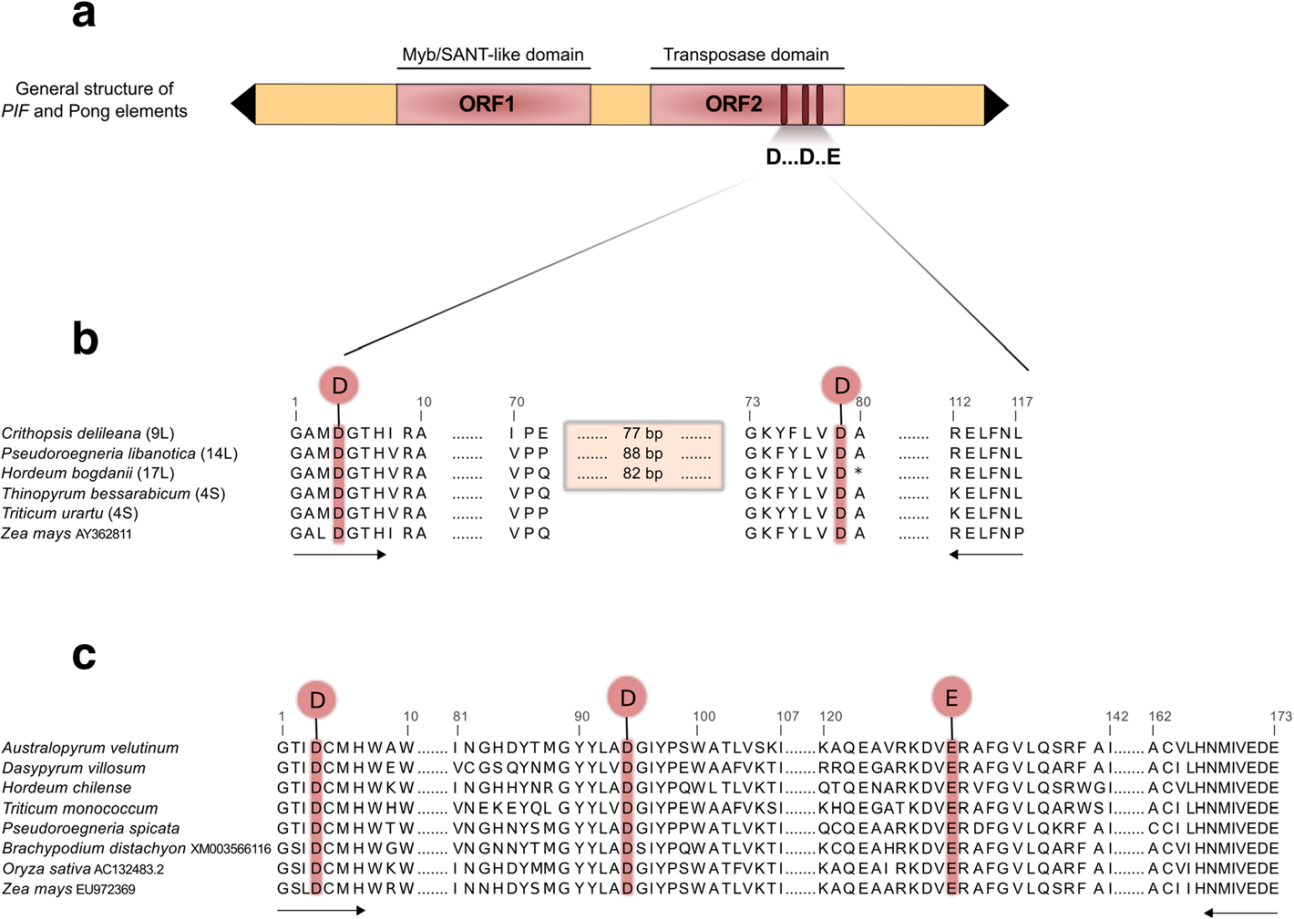
Structure of *PIF* and Pong-like elements. **a** General structure of *PIF* and Pong-like elements with the corresponding ORFs. ORF2 contains a “DDE” motif, a signature consisting of three conserved amino acids. **b** Comparison of the conserved *PIF* transposase domain with part of the “DDE” motif from five Triticeae samples and *Zea mays*. **c** Alignment of the conserved Pong transposase domain with the “DDE” motif. Triangles represent TIRs and rectangles represent ORFs. Grey rectangle indicates insertion. Primers used for amplification are indicated by bold arrows

Most *PIF* and Pong elements are approximately 4–6 kb long [28, 30, 35] and contain two open reading frames (ORFs), one encoding a transposase (ORF2), and one whose function is still not known (ORF1) (Fig. 1a), though it is thought to be involved in DNA binding activity and protein-protein interactions [39–42]. The transposase contains a “DDE” motif, a signature consisting of an amino acid triad identified in the transposases of most DNA transposon superfamilies (Fig. 1a) [43, 44]. The “DDE” motif consists of two aspartic acid (D) residues and glutamic acid (E) residue interspersed within a relatively well conserved domain of amino acids, which has been used to establish the evolutionary relationships among *PIF* and Pong elements [35, 37, 38, 42, 45, 46]. In some *PIF* elements the transposase gene is interrupted by between one to three insertions characterized as introns [36, 38, 47].

We have demonstrated that *PIF* and Pong elements in the genomes of diploid Triticeae species are abundant and highly variable, and represent multiple diverse lineages within genomes that appear to predate the origin of the tribe itself [45, 46]. To determine whether they are transcriptionally active, we screened 15 diploid individuals from 13 species for the presence of *PIF* and Pong-like transcripts, and we performed phylogenetic analyses of both genomic DNA and cDNA copies to establish whether the detected transcripts are produced by several or only few transposase lineages. We found that *PIF*-like transposases are actively transcribed in Triticeae and that most, if not all, transposase lineages that we previously identified are transcriptionally competent [45]. In contrast to our evidence of *PIF* transcription, we did not detect any transcriptional activity of Pong elements in any sample.

## Methods

### Plant material

**Fig. 2:**
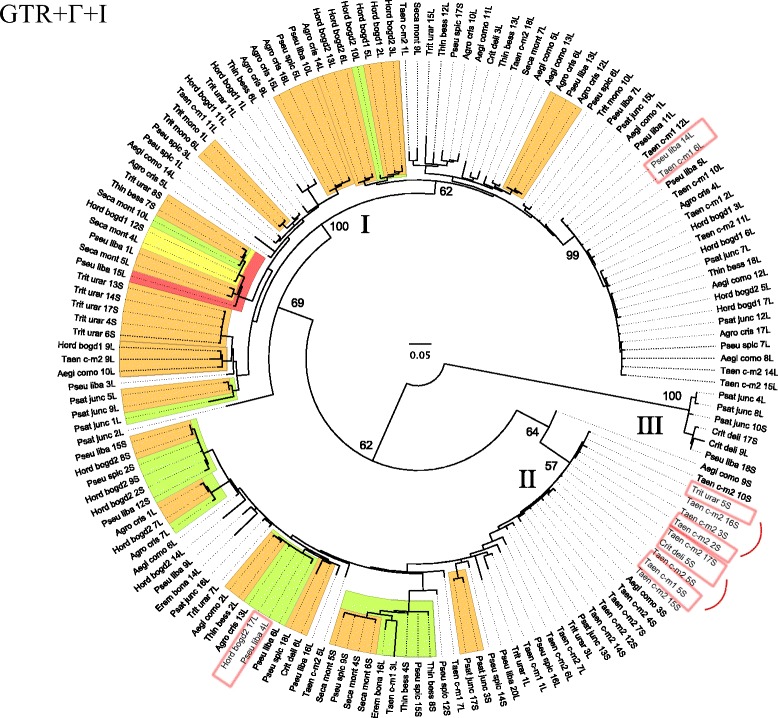
100-replicate ML bootstrap analysis of 156 *PIF-*like cDNA transposase sequences from Triticeae under the GTR+Γ+I model of evolution. Colored clades represent clades with bootstrap support above 80%. Bootstrap values of main clades are displayed with numbers. Red rectangles indicate identical sequences from distinct genera. Taxon labels combine the first four letters of the genus and species names. Numbers following taxon names distinguish cloned sequences from within individuals and are consistent among Figs. 2 and 3. S designates short sequences without the intron; L designates long sequences with the intron

**Fig. 3:**
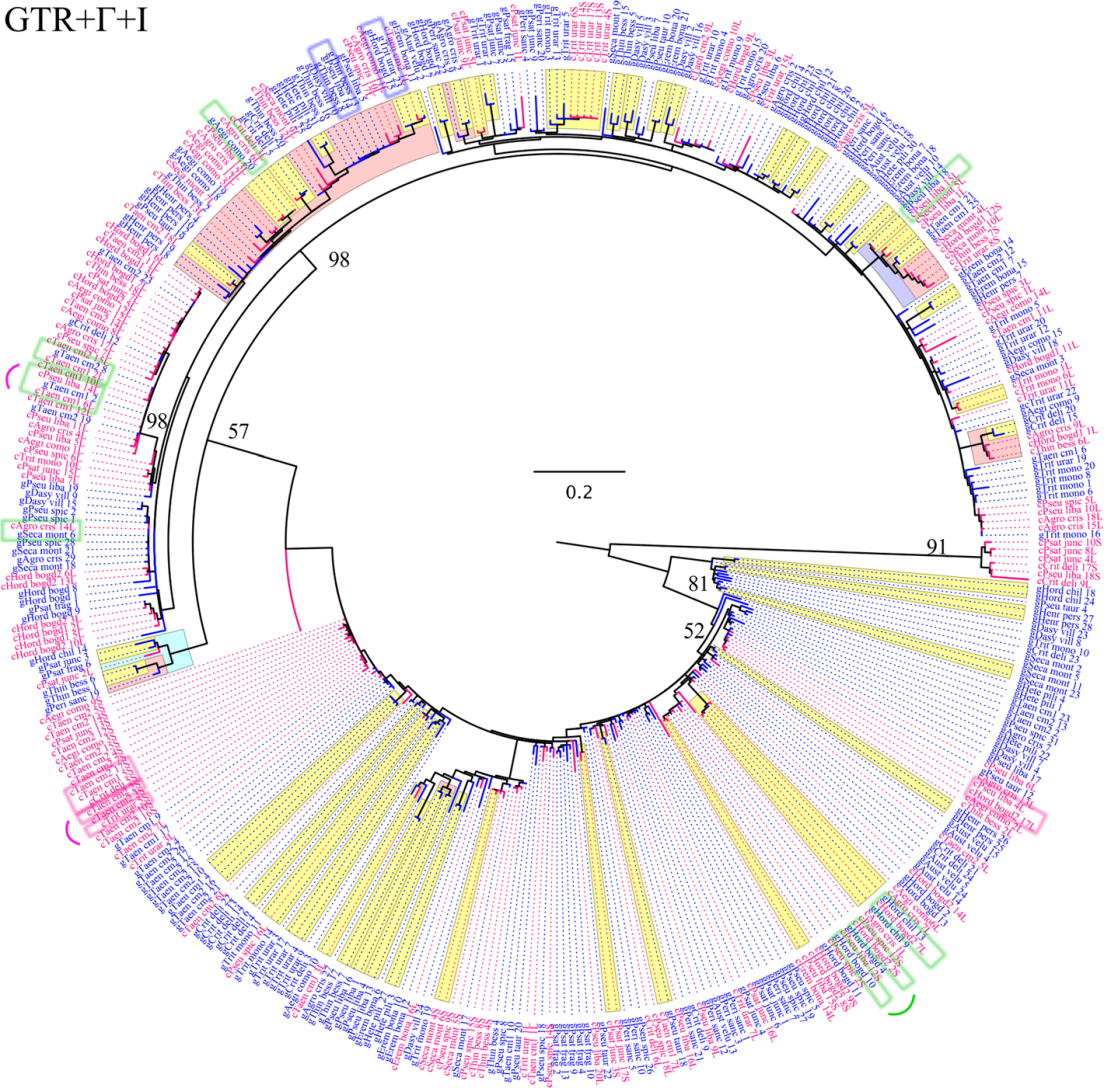
100-replicate ML bootstrap analysis of 156 *PIF-*like cDNA transposase sequences (c-pink) and 240 genomic (g-blue) *PIF* transposase fragments under the GTR+Γ+I model of evolution. Colored clades represent clades with bootstrap support above 80%. Bootstrap values of main clades are displayed with numbers. Pink rectangles indicate identical cDNA sequences; blue rectangles indicate identical genomic DNA sequences; green rectangles indicate identical cDNA and genomic DNA sequences. Taxon labels combine the first four letters of the genus and species names. Numbers following names distinguish cloned sequences from within individuals and are consistent among Figs. 2 and 3. S designates short sequences without the intron; L designates long sequences with the retained intron

Fifteen accessions of 13 species representing 11 Triticeae genera were used to survey for the presence of *PIF* and Pong-like related transcripts (Table 1). To avoid the potentially confounding phylogenetic effects of auto- and allopolyploidy, only diploid taxa were chosen for this study. Seeds were obtained from the USDA and have associated chromosome counts. All plants were grown at the University of Illinois at Chicago greenhouse under common conditions. The *PIF* cDNA sequences were analyzed alone (Fig. 2) and in combination with two hundred and forty genomic *PIF* ORF2 sequences from 22 diploid Triticeae samples [[45]; Table 1] (Fig. 3).

**Table 1:** List of Triticeae taxa included in *PIF* transcriptional analyses. Samples are represented with their names and collection numbers

	genomic *PIF* ^a^	cDNA *PIF*
Species name	Sample source and reference number	Sample source and reference number
*Aegilops comosa* Sibth. & Smith	USDA/G602	USDA/PI 542175
*Agropyron cristatum* (L.) Gaertn.	USDA/PI 279802	USDA/PI 439925
*Australopyrum velutinum* (Nees) B.K.Simon	USDA/D 2873–2878	
*Crithopsis delileana* (Schult.) Roshev.	USDA/H 5562	USDA/H 5562
*Dasypyrum villosum* (L.) P.Candargy	USDA/D 2990	
*Eremopyrum bonaepartis* (Spreng.) Nevski	USDA/PI 227344	USDA/PI 219970
*Henrardia persica* (Boiss.) C.E.Hubb	USDA/H 5556	
*Heteranthelium piliferum* (Banks & Sol.) Hochst.	USDA/PI 402352	
*Hordeum bogdanii* Wilensky (1)		USDA/PI 531760
*Hordeum bogdanii* Wilensky (2)	USDA/PI 531762	USDA/PI 531762
*Hordeum chilense* Roem. & Schult.	USDA/PI 531781	
*Peridictyon sanctum* (Janka) Seberg, Fred., & Baden	USDA/KJ 248	
*Psathyrostachys fragilis* (Boiss.) Nevski	USDA/PI 343192	
*Psathyrostachys juncea* (Fisch.) Nevski	USDA/PI 206684	USDA/PI 272136
*Pseudoroegneria libanotica* (Hack.) D.R.Dewey	USDA/PI 228391	USDA/PI 228389
*Pseudoroegneria spicata* (Pursh) Á.Löve	USDA/D 2844	USDA/PI 236672
*Pseudoroegneria tauri* (Boiss. & Balansa) Á.Löve	USDA/PI 401319	
*Secale montanum* Guss.	USDA/T 36554	USDA/T 36554
*Taeniatherum caput-medusae* (L.) Nevski (1)	USDA/PI 208075	USDA/PI 222048
*Taeniatherum caput-medusae* (L.) Nevski (2)	USDA/PI 283240	USDA/PI 220591
*Thinopyrum bessarabicum* (Săvul. & Rayss) Á.Löve	USDA/PI 531711	USDA/PI 531711
*Triticum monococcum* L.	USDA/PI 221413	USDA/PI 10474
*Triticum urartu* Tumanian ex Gandilyan	Morrison s.n.	Morrison s.n.

^a^Genomic *PIF* sequences are from [45]

### DNA, RNA extractions and cDNA synthesis

The DNA was extracted for previous phylogenetic studies from fresh or dried leaf material, using a CTAB-based method [48]. RNA was extracted from fresh leaf material harvested from reproductively mature plants (Table 1). Plant tissue was snap-frozen in liquid nitrogen and total RNA was extracted using a commercial extraction kit (Promega), following the manufacturer’s instructions. Crude total RNA preparations were treated with TURBO DNA-free™ (Ambion) to remove residual DNA. RNA quality was inferred by running 5 μl on an agarose gel, and RNA concentrations were estimated using a NanoDrop Spectrophotometer (Thermo Fisher Scientific Inc., MA, USA). Prior to cDNA synthesis, the presence/absence of genomic DNA contamination was tested for all RNA preparations using PCR reactions with *PIF* and Pong primers known to work on genomic DNA, and an RT- control supplied with the cDNA synthesis kit. cDNA was generated from DNA-free total RNA using the Protoscript RT-PCR kit (NEB) using 2 μl of random and oligo-dT primers and following the manufacturer’s protocol. The cDNAs were used as templates in amplification reactions as described below.

### Amplification of the *PIF* ORF2 conserved domain

Triticeae-specific degenerate primers (c*PIF*-for: GGAGCHWTNGATGGYACWCAC, c*PIF*-rev: AAGGTTGAAYAGCTCCYT) targeting a conserved portion of the *PIF* transposase were used for all PCR amplifications (Fig. 1b). These primers are anchored in two highly conserved amino acid residue motifs (GAMDGTH and RELFNL respectively) of the transposase gene, surrounding the “DD” portion of the “DDE” motif. The predicted TPases encoded by plant *PIF* transposons vary in length from 392 to 432 amino acids [42]; the amplified portion represents between 120 and 147 amino acids. The position of the “DD” transposase fragment was predicted by comparison of a reduced set of aligned Triticeae sequences to the corresponding portion of the “DDE” motif from a *Zea mays PIF* element (AY362811; Fig. 1b). All amplifications were carried out in a 10 μl reactions containing 50 ng of cDNA, 10× PCR buffer, 0.1 mmol/L of each primer, 0.5 units of Taq polymerase (Sigma), 0.2 mmol/L of each dNTP, and 1.5 mmol/L MgCl_2_. The PCR amplification conditions were: 5 min of DNA denaturation at 95 °C, followed by 35 cycles of 30 s at 95 °C, 45 s at 57 °C and 60 s at 72 °C for each cycle. The last cycle was followed by a 10 min final extension at 72 °C.

### Amplification of the Pong ORF2 conserved domain

Degenerate primers (Pong-for: GGCWCCATYGAYTGTATGCAC, Pong-rev: YTCGTCYTCVACYATCATRTTGTG; [37]) were used for cDNA amplification of approximately one-third or 520 bp of conserved region of the Pong transposase domain, including the “DDE” motif. These primers are anchored in two highly conserved amino acid residue blocks (GTIDCMH and NMIVEDE) of the transposase gene (Fig. 1c) and were previously shown to work on genomic DNA [37, 46]. All amplifications were carried out as described in [46].

### Cloning, sequencing and sequence alignment

PCR products were cloned prior to sequencing, and multiple clones from each species were sequenced to evaluate intra-individual transposase diversity. Three PCR reactions were run for each cloning reaction to counter the potential effects of PCR drift [49]. PCR products from replicated reactions were isolated on 1% agarose gels, combined and purified on columns (Qiagen). Cleaned products were cloned into pGEM-T Easy vectors (Promega) and transformed into *E.coli* JM109 competent cells (Promega) according to the manufacturer’s instructions, except that all reactions were halved. Positive (white) colonies containing the insert were PCR amplified as described above. The resulting fragments were cleaned with 0.2 μl exonuclease and 0.4 μl shrimp alkaline phosphatase, and sequenced in both directions with the PCR primers. Sequencing was performed on an ABI 377 automated sequencer (Applied Biosystems). The nucleotide and inferred amino acid sequences of *PIF*-like transposases were aligned using CLUSTALW [50] with default parameters, and then manually adjusted in MacClade 4.08 (Maddison and Maddison). All alignments are available upon request.

### Phylogenetic analysis

Phylogenies were estimated using maximum parsimony (MP) and maximum likelihood (ML). Parsimony analyses and pairwise sequence distances were estimated with PAUP* v.4.0b10 [51]. The parsimony bootstrap method, with 1000 replicates with heuristic search, was used to estimate the robustness of the clades [52] (tree not shown). For the ML analysis, the appropriate model of sequence evolution was determined by jModelTest [53–55] and the corrected Akaike information criterion [56]. The selected models of evolution were implemented in the Mac OS X version of GARLI v.0.95 [57] for analysis. Following the recommendations of the author, multiple (50) analyses with random starting tree topologies were performed for each data set. Runs were set for an unlimited number of generations, and automatic termination following 10,000 generations without a significant change in topology. Bootstrap support for each tree was estimated based on 100 ML bootstrap replicates with the same options used to generate the ML tree. All sequences were deposited in the NCBI GenBank database (accession numbers MF281799-MF281954).

## Results

### Isolation and characterization of *PIF* cDNAs

We isolated, cloned, and sequenced 156 unique cDNA fragments from the conserved transposase domain of *PIF*-like TEs in 15 diploid Triticeae samples. As in our previous analysis of genomic *PIF* sequences, all fragments corresponded to the “DD” portion of the “DDE” transposase motif (Fig. 1b) [45]. PCR amplifications yielded two bands of approximately 360 and 440 bp, labeled “S” (short) and “L” (long) in the *PIF* phylogenies (Figs. 2 and 3), for all samples except *Eremopyrum bonaepartis*, *Triticum monococcum*, and *Agropyrum cristatum*, in which only the longer fragments were detected. The 156 cDNA sequences revealed that the length difference between long and short fragments is explained by the retention of an intron during transcription by 112 *PIF* transposase fragments, ranging in size from 72 to 88 bp. The intron was located six residues upstream of the second D (Fig. 1b), and contained a stop codon in 85 of the sequences. Approximately 30 of the 156 products contained additional deletions and insertions of one or a few bases; thus, some apparently non-functional gene copies are being transcribed. Sequences showed 58.65–100% nucleotide identity, with the highest level of divergence (41.35%) found between *E. bonaepartis* 14L and *Psathyrostachys juncea* 8L. Identical ORF cDNA fragments were detected in different samples in four cases (marked with rectangles on Fig. 2): *Pseudoroegneria libanotica* 14L and *Taeniatherum caput-medusae*1 6L; *P. libanotica* 4L and *Hordeum bogdanii*2 17L; *T. urartu* 5S and *T. caput-medusae*2 2S; and *T. caput-medusae*2 17S, *T. caput-medusae*1 5S, and *Crithopsis delileana* 5S.

### Triticeae contain transcriptionally active *PIF*, but not Pong elements

Our results show that *PIF* is actively transcribed in all samples. We did not detect any transcriptional activity for the closely related Pong elements, even though a previous study of Pong genomic sequences [46] showed that Pong sequences are, like *PIF*, widely dispersed within Triticeae, with multiple distinct and genetically diverse transposases coexisting within individual genomes. We do not think the lack of transcripts can be explained as a technical artifact due to poor amplification, because the amplification primers used here are the same ones used to successfully amplify a wide diversity of genomic Pong sequences from the same Triticeae species [46]. The Pong results not only further highlight a difference in transcriptional activity between these otherwise very similar groups of elements, but they also served a practical purpose, as an additional control confirming the absence of genomic DNA contamination in all RNA preparations.

### Phylogenetic analysis

All phylogenetic analyses of the Triticeae *PIF*-like cDNAs were performed on a region of approximately 360 bp coding sequence; the intron was excluded because of alignment ambiguities. Maximum parsimony topologies (not shown) were in general accordance with the ML topologies, but there was more resolution and support in the ML trees. Given the difficulties of finding an outgroup while providing clarification of phylogenetic relationships between TEs, we used the mid-point rooting method [58] for all of the phylogenetic trees. Although *PIF* sequences from grass genera outside the wheat tribe are available, they are not appropriate as outgroups for the Triticeae elements because the *PIF*-like lineages within Triticeae appear to predate the tribe’s origin [i.e., some *PIF* elements from within the Triticeae are more closely related to grass elements from outside of the tribe than they are to other elements from within the tribe [[45]; see also [38, 42]].

#### *Phylogeny of* PIF *cDNA transcripts in Triticeae*

This data set included all 156 cDNA fragments from all 15 accessions. (A phylogeny of 44 cDNA *PIF* transcripts with no intron is presented as an Additional file 1). The best topology (−lnL = 5,169.16730; Fig. 2) revealed three main groups of *PIF* cDNAs in Triticeae (I-III in Fig. 2). *Psathyrostachys juncea* 2L was sister (69% bootstrap support) to group I (100% bootstrap), which was the largest and the most complex group, and was further subdivided into two weakly supported subgroups. Group I contained sequences from all samples except *E. bonaepartis*. Within this group, *P. libanotica* 14L was identical to *T. caput-medusae*1 6L (indicated with rectangles on Fig. 2). Group II (weakly supported) was represented by sequences from all samples except *H. bogdanii*1 and *T. monococcum*. Within this group, three sets of sequences were identical: *H. bogdanii*2 17L and *P. libanotica* 4L; *T. caput-medusae*2 2S and *T.urartu* 5S; and *C. delileana* 5S, *T. caput-medusae*2 17S, and *T. caput-medusae*1 5S (indicated with rectangles on Fig. 2). *Aegilops comosa* 9S was sister to group II with bootstrap of 64%. Group III (100% bootstrap support) only included sequences from *C. delileana*, *P. libanotica*, and *P. juncea*. The small size of this group points to a combination of differences in element transcriptional activity, the loss of some lineages through stochastic events and natural selection, and/or random sampling artifacts. *Crithopsis delileana* and *P. libanotica* exhibited very broad distribution, with cDNA sequences in all of the main evolutionary lineages identified.

#### *Phylogeny of genomic and cDNA* PIF *transposase fragments*

This analysis included 156 cDNAs generated for this study along with 240 genomic *PIF* sequences from a previous phylogenetic study of *PIF* sequences in Triticeae [45] (Fig. 3). Of the 240 genomic sequences, 113 had frameshifting indels or stop codons, and thus are probably not functional. The best topology (−lnL = 11,008.92254; Fig. 3) revealed multiple distinct transposase cDNA fragments grouped with genomic sequences in well-defined and generally well-supported clades (Fig. 3). The wide distribution of cDNA sequences among the genomic sequences showed that they are derived from multiple evolutionary lineages, indicating that distinct transposases have retained transcriptional competence during the evolution of the tribe and have the potential to function simultaneously within a genome (Fig. 3).

Eight cDNAs were identical to genomic *PIF* fragments (indicated with green rectangles in Fig. 3), suggesting that they originated from identical or nearly identical transposase fragments (although only half of them are paired with genomic copies from the same species). Of these eight transcripts, seven were derived from transposases with no frameshifting indels or stop codons. The eighth, c*H. bogdanii*2 7L, is characterized by four single base pair deletions, resulting in a change of the reading frame, thus demonstrating that transcriptional activity does not necessarily indicate functional activity. Two pairs of genomic transposase sequences were identical (marked with pale blue rectangles on Fig. 3): g*Thinopyrum bessarabicum* 8 and g*P.libanotica* 14; and g*H. bogdanii* 15 and gT*. urartu* 23.

## Discussion

Transcription is the first of several steps required for TE transposition [59]. Autonomous elements (i.e. elements that encode all functional products required for transposition) have the potential to self-activate or regulate the activity of related non-autonomous versions, which are ubiquitous in grass genomes [11]. To ensure the viability of their host, and therefore their own survival, optimized transmission and restricted transpositional activity are the hallmark of many TE families. Once integrated in the host genome, TEs rapidly accumulate small insertions, deletions, and rearrangements that alter their structural integrity and render them inactive [23, 25, 26]. Plant cells have also developed a variety of transcriptional and post-transcriptional regulatory mechanisms to protect their genomes against TE movement, including silencing by increased DNA methylation of promoter regions, histone modifications, or small RNA interference [19, 60–62].

### Transcriptional activity of *PIF* and Pong-like TEs in Triticeae

Our work on the evolutionary dynamics of *PIF* and Pong transposase activity in Triticeae had two major goals. The first was to determine whether *PIF* and Pong are transcriptionally active in Triticeae, and the second was to assess the diversity of transcribed transposase lineages. We found that *PIF*-like transcripts are present throughout the Triticeae, indicating that they have remained transcriptionally active throughout of the long history of the tribe (13–25 mya; [63]). Phylogenetic analysis of both genomic DNA and cDNA revealed that the detected *PIF* transcripts belong to distinct clades, and that most, if not all transposase lineages have remained transcriptionally competent. In contrast, we did not detect any transcriptional activity of Pong elements in any sample, in spite of previous work [46] showing that the diversity of Pong elements in Triticeae genomes is comparable to that of *PIF* elements in the same species, with multiple distinct lineages coexisting within a single genome. Although this work is focused on TE transcriptional activity in mature leaf material only, the lack of Pong activity in the wheat tribe also contrasts with observations from other plant species; Pong elements have undergone recent amplification in *Arabidopsis* and *Brassica* [36], and are transcriptionally active in rice [30, 64–66]. One plausible explanation for the lack of Pong-related transcription within Triticeae genomes could be the failure of a related or unrelated TE, transposase gene, or mechanism to activate the transcription machinery of Pong elements in a common ancestor of Triticeae. It is highly unlikely that individual Pong copies have been transcriptionally inactivated separately due to natural selection and/or genetic drift. Based on our previous analyses of Pong elements within Triticeae genomes, their expansion seems to be recent [46], thus it is possible that the element is still active but another mechanism has failed to instigate its transcription and therefore activity.

### Phylogeny of *PIF* cDNA transcripts

Genome-wide studies of transcriptional activity of 56 maize TE families, the *PIF* family included, have demonstrated that TE Expressed Sequence Tags (ESTs) are located only in a few clades of genomic sequences, indicating that few evolutionary branches of the TEs are transcriptionally active [67]. However, in contrast to these findings, our results revealed that the majority of *PIF* lineages have retained transcriptional capacity.

The wide distribution of distinct taxa in groups I and II in Fig. 2 suggests that diverse ancestral lineages were vertically transmitted and have remained transcriptionally active during the evolution of the tribe (13–25 mya; [63]). Elements from groups I and II are missing from only a few individuals; this could be attributed either to loss from those genomes or to a sampling artifact. Group III (Fig. 2) is represented in far fewer individuals, which may be due to differential evolutionary success of this transposase lineage due to selection, and/or to stochastic losses. However, the presence of these transcripts in species derived from basal branches of the wheat tribe such as *Psathyrostachys* [68, 69] indicates this lineage was already present at the beginning of Triticeae radiation, and later lost from some of the descendants.

The presence of identical *PIF* transposase fragments shared across species boundaries suggests that recent or ongoing occasional horizontal transfer (HT) events have played a significant role in the complex distribution of *PIF* elements in Triticeae. This was also supported by our previous analysis of *PIF* dynamics in Triticeae [45], in which we identified two pairs of genomic *PIF* transposase gene fragments that exhibited extremely high nucleotide sequence identities (marked with pale blue rectangles on Fig. 3). Triticeae genera diverged 13–25 mya [63], and it is highly unlikely that their transposase sequences diverged at the same time as the hosts and maintained such high sequence similarity, even if they are under selective constraints [45]. Here, the identification of identical pairs of cDNA and genomic transposase fragments provides further evidence that HT plays a role in the distribution of *PIF* elements among genera.

## Conclusion

*PIF* and Pong-like elements are widely dispersed within the genomes of diploid Triticeae species. However, both TE families display unique features and vary considerably in their transposase transcriptional activity. No Pong-related transcripts were detected, while an abundance of diverse *PIF*-related transcripts were identified in all samples, indicating wide variations in the activity of closely related transposon families within the same genome. Multiple distinct transcriptionally competent *PIF* transposase clades were discovered, revealing that transcription of *PIF* elements in Triticeae is not restricted to few evolutionary lineages.

## Additional file

Additional file 1: 1000-replicate ML bootstrap analysis of 44 *PIF*-like cDNA transposase sequences with no intron from Triticeae (only bootstrap values above 50% are shown). Red rectangles indicate identical sequences from distinct genera. Numbers following taxon names distinguish individuals within species and numbers in parentheses distinguish cloned sequences from within individuals. (EPS 1457 kb)
